# Felt sense dynamics: a practice-based framework for tracking the arc of somatic emotional processing

**DOI:** 10.3389/fpsyg.2026.1873403

**Published:** 2026-08-06

**Authors:** Naomi Sanderovsky

**Affiliations:** University of Maryland Global Campus, Adelphi, MD, United States

**Keywords:** body psychotherapy, emotional processing, felt sense, felt sense dynamics, grief, nonduality, organic inquiry, pendulation

## Abstract

When a felt sense is genuinely attended to, it responds. This paper introduces Felt Sense Dynamics, a phenomenological, practice-based framework that names and maps that response across five recurring phases: Steadying, Strengthening, Spreading, Shifting, and Softening, collectively referred to as the Five S’s. Gendlin’s concept of the felt shift names the destination of somatic processing; Felt Sense Dynamics attends to the journey: the phenomenological arc of sensation between initial contact and that arrival. The framework was developed inductively through sustained observation across several years of somatic practice in grief retreats, coaching, and practitioner training contexts, notably including many licensed therapists, mental health professionals, and somatic practitioners. The framework identifies these phases as observable tendencies that have appeared repeatedly in the author’s practice-based observations when sensation is held with compassionate curiosity without attachment. A distinguishing orientation of the framework is its aim toward participant self-sufficiency. Many somatic models function as roadmaps for the practitioner, organizing the therapeutic encounter through framing, naming, and directed technique. Felt Sense Dynamics cultivates in participants a transferable capacity for compassionate self-witnessing. The practitioner’s role is to teach this capacity, not to be its necessary condition. The paper situates the framework in relation to established somatic modalities, including Gendlin’s Focusing, Levine’s Somatic Experiencing, Hakomi, Polyvagal Theory, Sensorimotor Psychotherapy, and EMDR, and in explicit dialog with five adjacent frameworks: Winhall’s Felt Sense Polyvagal Model, Selvam’s Integral Somatic Psychology, Fosha’s AEDP four-state map, the Community Resiliency Model’s Shift and Stay skill, and the somatic reappraisal framework, whose empirical findings this paper extends phenomenologically. The facilitation protocol, including grounded preparation, memory activation, macro-pendulation, and pleasure anchoring, is described in full. The paper is grounded epistemologically in organic inquiry and closes with a preliminary observational structure and directions for future empirical investigation.

## Introduction

The body knows things before language does. This is the foundational insight of somatic psychology: that below thought, below narrative, below the names we give our states, there is a pre-verbal, holistic knowing that precedes and exceeds cognitive formulation. Gendlin (1978) named this the felt sense and identified its destination: the felt shift, the moment of bodily resolution that marks the completion of somatic processing. What he offered was a landmark. What remained to be mapped was the terrain between arrival and that landmark: what happens, phase by phase, in the body when a felt sense is genuinely attended to with patient, non-attached awareness. Felt Sense Dynamics is a framework for that terrain.

Developed inductively through sustained observation across several years of somatic practice in grief retreat work, grief accompaniment, and practitioner training, the framework identifies five recurring phases in the trajectory of sensation during emotional processing: Steadying, Strengthening, Spreading, Shifting, and Softening. These phases are not stages to be achieved or directed but tendencies that have appeared repeatedly in the author’s practice-based observations when sensation is held with compassionate curiosity without attachment. Critically, the framework was developed not as a theoretical proposition derived from the literature but as an empirical observation derived from practice. It is a pattern that presented itself repeatedly across sessions and subsequently sought theoretical articulation.

Notably, many participants across these contexts were themselves licensed therapists, mental health professionals, and somatic practitioners, providing an informal but substantive stress-test of the framework’s coherence and resonance within clinically trained populations. While the framework draws on and is in dialog with the psychotherapeutic literature, it is offered as a resource for somatic practitioners, coaches, grief educators, and facilitators, as well as for licensed therapists who may find it useful. The Five S’s describe a phenomenon that has appeared across all these practice contexts. The facilitation protocol is designed to be adapted to each context.

The paper proceeds in four parts. The first situates Felt Sense Dynamics within the existing somatic landscape and in relation to five adjacent frameworks, including Winhall’s Felt Sense Polyvagal Model, Selvam’s Integral Somatic Psychology, and Fosha’s AEDP, whose distinctions clarify the framework’s contribution. The second describes the framework in detail, including the Five S’s and the facilitation protocol in which they are embedded. The third addresses theoretical questions including dissociation, trauma-informed structure, and the framework’s non-dual orientation. The fourth discusses the epistemological basis in organic inquiry and proposes directions for future empirical investigation.

## Practice-based origins and development of the framework

Felt Sense Dynamics did not begin as a theoretical proposition. It began as a recurring observation that resisted being ignored.

The author’s background in somatic psychology included training in several established modalities, each of which offered practitioners a way to locate, name, and work with bodily sensation. What they shared, in the author’s observation, was a characteristic stopping point: the practitioner would guide a participant to identify where a feeling lived in the body and then proceed to interpretation, regulation, or the next intervention. The sensation had been contacted, but it had not been fully accompanied.

Training in non-dual therapy with Georgi Y. Johnson and Bart ten Berge, whose approach emphasizes non-directed witnessing of whatever arises in awareness, offered a different orientation: stay with what is present, without guiding, framing, or resolving it. Applying this stance to somatic work with grief retreat participants and individual sessions, the author began noticing something consistent. When participants were invited simply to remain with a sensation, without being guided toward any particular outcome, the sensation itself began to move; and to move in recognizable ways.

The language came from participants before it came from the author. In the early seconds they would say, “It’s still there,” holding steady. As attention was sustained, this became “It’s getting stronger,” or “It’s more intense now.” A little later: “It’s moving, spreading outward,” or “Now it’s jumped to my throat.” Then, “Something shifted; it feels different, like it’s pulsing now, or tingling.” Finally, there was a settling: “It’s fading,” “It’s softer now,” or “It is dissipating.” These were not words the author had offered; they were the body’s own report.

Over many sessions, across varied emotional content and participant backgrounds, the pattern recurred. The author experimented with other organizational schemes—fewer phases, more phases, different sequencing—but none captured the observations as parsimoniously. The five-phase topology emerged as the structure that best described the recurring transitions without imposing artificial boundaries on what was, in practice, a fluid continuum. The alliterative naming—Steadying, Strengthening, Spreading, Shifting, and Softening—came afterward as a mnemonic to help participants recognize and remember what they had experienced. Notably, the arc appeared whether participants had been taught the Five S’s in advance, consistent with the possibility that the topology was describing something intrinsic to the process rather than being produced by the expectation of it.

An important point of convergence came through discussion with Johnson (2017), author of non-dual therapy, who confirmed that the phenomenological trajectory the author had observed corresponded with her own extensive experience working somatically with sensation. This cross-practitioner resonance suggested that the pattern was not an artifact of the author’s specific facilitation style.

The framework also drew repeated informal testimony from experienced somatic practitioners who participated in retreat and training contexts. Several independently described the same recognition: that this was “the missing piece” in their somatic work; an account of what happens when sensation is not redirected or interpreted but simply accompanied through its full arc. Others articulated what participants without somatic training frequently expressed more directly: *“So that is how you feel a feeling.”* Another common reflection was, “How strange that we were never taught how to fully feel a feeling.” These responses, offered across many contexts and by clinically trained participants positioned to notice inconsistencies, led the author to regard the topology as descriptively adequate rather than theoretically imposed. These consultations were informal and practice-based rather than part of a structured collaborative research design, and they are therefore presented here as corroborating impressions rather than systematic data.

Observations were documented contemporaneously through practitioner session notes tracking participant-reported sensation descriptors: location, movement, intensity, temperature, texture, and reported shifts. Transitions between phases were noted when participants spontaneously described qualitative changes in these descriptors without prompting. The purpose of this article is therefore not to establish the empirical validity of the Five S’s, but to articulate a phenomenological framework with sufficient conceptual clarity to permit systematic empirical evaluation.

## Situating felt sense dynamics in the somatic field

### Foundations: Gendlin and the felt sense

Gendlin’s (1978, 1996) focusing established that the body holds meaning in a pre-linguistic form: a felt sense that is holistic, vague, yet distinctly felt, and that carries implicit knowing about a situation or experience. The core movement of focusing is toward the felt shift: the moment when an accurately chosen word, image, or phrase causes the body to release, relax, or open in recognition. As Gendlin (1978, p. 35) described it, this shift may be “only a slight release,” but it is real and always more than intellectual understanding.

Gendlin’s attention was primarily on the moment of resonance and the body’s response to accurate language. The present framework attends to what occurs prior to and between such moments: the movement of sensation itself, independent of language. The question it asks is not ‘what does this felt sense mean?’ but ‘how does this felt sense unfold?’ How does this feeling fully express itself from first contact, through its peak, and into its natural waning? What is the felt sense feeling as sensation lives, breathes, and moves in the body? This is a fundamental reorientation: from felt sense as a holder of meaning to felt sense as a dynamic process. The Five S’s describe that process with a specificity that Gendlin’s framework, with its focus on the semantic dimension of somatic experience, leaves open.

### Adjacent framework: Winhall’s felt sense polyvagal model

Winhall’s (2021) Felt Sense Polyvagal Model (FSPM) integrates Gendlin’s Focusing with Porges’ Polyvagal Theory, mapping felt sensing onto four autonomic states. These broadly correspond to ventral vagal connection (Flock), sympathetic activation (Fight/Flight), dorsal collapse (Fawn), and freeze, plotted on axes of attachment and arousal. The practitioner uses this graphic framework to identify where a participant’s nervous system is positioned and to support movement toward greater regulation and connection. The FSPM’s orienting question is: from which autonomic state does a felt sense arise, and toward which state can it move?

Felt Sense Dynamics shares with FSPM a grounding in both Gendlin and Polyvagal Theory, and both frameworks hold the body as the primary site of knowing. The distinctions, however, are significant. FSPM is a practitioner-held framework: the clinician uses the graphic model to identify where the participant’s nervous system is positioned and to guide movement toward greater regulation. It is, by design, a map for the guide. Felt Sense Dynamics inverts this orientation. The Five S’s are not a map the practitioner consults; they are a capacity the participant learns to inhabit. FSPM asks: from which autonomic state does a felt sense arise, and toward which state can it move? Felt Sense Dynamics asks simply: how does this felt sense unfold when it is genuinely accompanied, without naming, framing, or directing? FSPM maps the nervous system context of felt sensing; Felt Sense Dynamics provides a phenomenological account of what sensation itself does when met with sustained, non-attached presence. The two frameworks are complementary and address adjacent dimensions of the same somatic process. Both should be cited together in any review of felt-sense-based approaches.

### Adjacent framework: Selvam’s integral somatic psychology

Selvam’s (2022) Integral Somatic Psychology™ (ISP) offers a published seven-step and companion four-step protocol for the embodiment of emotion. Selvam’s core premise, derived from his training with Levine and from his own clinical observations, is that emotions confined to a limited region of the body are more dysregulating than emotions that are allowed to expand into additional regions. The ISP emotional embodiment protocol involves a practitioner actively guiding a client to locate an emotion in one area of the body, locally expand it, locate it in a second area, expand it there, and then pivot to physiological markers of integration.

The ISP protocol’s attention to expansion across body regions shares a surface resemblance with the Spreading and Shifting phases of the Five S’s, in that both observe emotion moving from a localized site to a wider field within the body. The distinction, however, is fundamental on multiple levels. Selvam’s protocol is explicitly interventional: the practitioner directs the participant to expand the emotion to additional regions, operating on the premise that wider distribution increases regulatory capacity. Felt Sense Dynamics holds an entirely different premise, namely that sensation arrives where it does in the body for its own reasons, carrying its own intelligence. Grief that presents as a weight in the chest, anger that rises as heat along the back, or shame that contracts in the belly are not patterns to be redirected but expressions to be witnessed.

Each person carries a personal emotional geography, a unique somatic fingerprint for each emotional state, as individual as a person’s handwriting. Learning to read that geography is a profound form of self-knowledge and, over time, a source of self-regulation. When a person recognizes the heat rising up their back as an early signal of anger, they gain the opportunity to pause, feel its full arc, and choose their response rather than react. The Five S’s describe Spreading and Shifting as autonomous movements—what sensation does when accompanied by non-attached attention, without being directed anywhere. This contrasts intervention with witnessing, positioning the practitioner as navigator and the participant as learning to navigate themselves.

### Adjacent framework: Fosha’s AEDP four-state map

Fosha’s (2000) Accelerated Experiential Dynamic Psychotherapy (AEDP) describes a four-state phenomenological map of the transformational process in therapy: State 1 (defenses and anxiety), State 2 (core affective experience), State 3 (core state, a quality of calm, perspectival openness, and unexpected ease), and State 4 (transformational affects and meta-therapeutic processing). AEDP holds that processing an emotion to completion involves moving through defense, into core affect, and through to core state, which is itself transformational. It uses somatic markers such as deep in-and-out breath and visible softening as guides to state transition.

AEDP’s State 3 (core state) shares experiential territory with the Softening phase of the Five S’s. Both describe a quality of spaciousness, release, and unexpected calm that arises when an affective process has been fully inhabited rather than avoided. The difference lies in the vehicle and in who holds the map. AEDP’s four-state framework is primarily a guide for the therapist, a way of tracking the therapeutic encounter and identifying interventions that might support the next transition. In AEDP, the framework is held by the practitioner, with co-regulation and attachment repair as the primary mechanisms of change. Felt Sense Dynamics takes a different approach. The Five S’s are not a framework held by the practitioner; they are a map the participant learns to read in their own body, independent of any therapeutic relationship. The witness can be a practitioner, a peer, or ultimately the participant. That is precisely the point: The goal is not a better therapeutic encounter but a transferable human capacity.

### Adjacent framework: Price and Hooven’s somatic reappraisal

Price and Hooven (2018) proposed the somatic reappraisal framework in a paper published in Frontiers in Psychology, arguing that sustained mindful interoceptive attention—attending to inner body experience with non-judgment and compassion—enables bodily sensations to become more granular and shift in ways that support new emotional meaning-making. Their framework describes a movement from implicit bodily sensation to insight that is “arising from and informed by” that sensation, rather than being “incongruently applied” to it.

Of all the adjacent frameworks considered in this paper, Price and Hooven’s somatic reappraisal work is the closest empirical neighbor. Their finding that sustained interoceptive attention causes sensation to become more granular, vivid, and shifting provides direct empirical grounding for the core observation of Felt Sense Dynamics: that sensation, when genuinely accompanied, moves. Price and Hooven attend to the cognitive and integrative outcomes of interoceptive attention—the meaning-making and reappraisal that emerge—Felt Sense Dynamics, however, provides the embodied account of the somatic arc through which those outcomes are reached. Price and Hooven empirically establish that something happens; Felt Sense Dynamics phenomenologically describes what that something looks and feels like as it unfolds, phase by phase, in the body. The relationship between the two frameworks is generative: their empirical foundation supports the present paper’s phenomenological contribution, and the Five S’s offer a descriptive structure that future research in the MABT tradition could use to operationalize and study the interoceptive arc more precisely.

### Trauma and regulation: Levine, Porges, and pendulation

Levine’s (1997, 2010) Somatic Experiencing (SE) introduced pendulation, the deliberate oscillation between activated sensation and regulated, resourced states, as the primary mechanism for trauma processing. SE holds that trauma is stored as incomplete survival responses in the nervous system and that titrated exposure allows those responses to complete and discharge. Porges’ (1994, 2011) Polyvagal Theory provides the neurophysiological scaffolding: the autonomic nervous system moves between ventral vagal states of social engagement, sympathetic activation, and dorsal vagal shutdown, and the work of regulation supports return to ventral vagal states.

Felt Sense Dynamics is consistent with both frameworks and incorporates macro-pendulation: rather than oscillating moment-to-moment within a single sensory experience, the facilitation protocol moves between multiple emotional experiences across a session, with regulated breathing as the return point between each. The session always concludes with somatic tracking of a pleasurable or positive feeling, ensuring the participant’s nervous system returns to a ventral vagal state before closing.

### Adjacent framework: the community resiliency model’s shift and stay

The Community Resiliency Model (CRM; Miller-Karas, 2021), a body-based self-care model developed from Somatic Experiencing and Gendlin’s Focusing, includes six emotion stabilization skills. One is “Shift and Stay,” which involves noticing when the body is in a state of discomfort or dysregulation and deliberately shifting attention to a more neutral or comfortable bodily sensation, then staying with that more comfortable sensation long enough for the nervous system to regulate.

The terminological similarity to elements of the present framework warrants explicit clarification. CRM’s Shift and Stay is a practitioner-directed regulation technique in which the client is guided to redirect attention from one sensation to another. It is an intervention in the body’s experience. Felt Sense Dynamics, by contrast, names Shifting and Softening as phases that occur naturally in a sensation already held with attention; they are descriptions of a trajectory, not instructions for redirecting it. The practice orientation is quite different: CRM’s Shift and Stay asks the client to move attention, whereas Felt Sense Dynamics observes that sensation moves on its own when genuinely accompanied.

### Presence and belief: Hakomi and sensorimotor psychotherapy

Kurtz’s (1990) Hakomi Method introduced mindfulness-based somatic tracking as a means of accessing unconscious beliefs stored in the body. The Hakomi therapist tracks micro-expressions, breath shifts, and postural changes, then uses gentle experiments to create new somatic experiences that challenge implicit core beliefs. Ogden et al.’s (2006) Sensorimotor Psychotherapy similarly foregrounds the body’s narrative: posture, gesture, and movement impulse reveal what verbal narrative cannot, and micro-movements can complete interrupted survival responses.

Felt Sense Dynamics incorporates elements of both. The quality of witnessing it cultivates reflects Hakomi’s loving presence. The framework also recognizes that movement impulses may arise spontaneously during the tracking process and can be followed rather than redirected, consistent with Sensorimotor Psychotherapy’s emphasis on allowing the body to complete what it began. It is worth noting that Kurtz’s Hakomi Method also includes a phenomenological cycle called the Sensitivity Cycle, comprising four stages: Insight/Clarity, Effective Action, Nourishment/Satisfaction, and Rest/Relaxation. This cycle tracks the organism’s movement through experience and toward effective action at the macro-experiential level, describing how a person moves through a complete episode of living. The Five S’s occupies a different register: intra-sensation phenomenology rather than macro-experiential cycling.

### Expressive release vs. witnessing: bioenergetic analysis

Lowen’s (1975) Bioenergetic Analysis (BA) offers a useful contrast that clarifies Felt Sense Dynamics by opposition. BA holds that emotional conflicts are stored in the body as chronic muscular tension, body armoring in Reich’s (1945) terminology, and that healing requires their active, expressive release through breathwork, grounding, movement, and vocalization. Participants in BA are guided to physically express suppressed emotions rather than to observe them.

Felt Sense Dynamics operates from a different premise. Sensation, in this framework, is not tension waiting to be released through expression but a dynamic process waiting to be witnessed into movement. The practitioner does not direct expression; they provide the quality of attention within which sensation can move on its own. This is not a rejection of expressive approaches but a different entry point: the question is not how to release what the body holds, but what happens when what the body holds is simply and fully seen.

### Bilateral integration: EMDR

Shapiro’s (2001) EMDR employs bilateral stimulation to integrate traumatic memories, with attention to bodily sensation as memories are processed. This framework is compatible with EMDR in principle, and with bilateral and rhythmic engagement. For example, slow side-to-side weight shifts or alternating knee taps could be incorporated when clinically appropriate. In the author’s own practice, sustained attention to sensation, including inviting participants to describe how “stuckness” itself lives in the body, has generally been sufficient to allow movement without routine use of formal EMDR techniques. This aligns with a broader convergence across somatic modalities on the value of both interoceptive attention and bilateral engagement for nervous system integration, while allowing practitioners to integrate EMDR according to their training and clinical judgment. The rhythmic side-to-side shifts and alternating taps described here are optional adjuncts left to practitioner discretion rather than required components of the framework. Clinicians such as Arielle Schwartz have raised important cautions about whether bilateral engagement reliably supports integration in all cases, an empirical question beyond this paper’s scope.

Table 1 summarizes key distinctions between Felt Sense Dynamics and the adjacent frameworks discussed above.

**Table 1:** Selected somatic and experiential frameworks and their relation to felt sense dynamics.

Framework	Primary question	Map held by	Evidence base	Primary distinction
Gendlin’s focusing	How does a felt sense shift when matched with accurate language?	Practitioner and participant	Phenomenological/Practice-based	FSD extends focus from the felt shift (destination) to the full arc of sensation preceding it.
Somatic experiencing (SE)	How can titrated activation and pendulation discharge stored trauma?	Practitioner	Clinical/Practice-based	FSD shares the emphasis on titration and safety but tracks the intra-sensory arc rather than survival-response completion.
Hakomi/Sensitivity cycle	How do mindfulness and experiments reveal implicit beliefs and cycles of experience?	Practitioner	Phenomenological/Practice-based	FSD adopts Hakomi’s mindful, loving presence but focuses on the movement of sensation itself, not belief restructuring.
Sensorimotor psychotherapy	How can movement and posture complete interrupted survival responses?	Practitioner	Clinical/Practice-based	FSD aligns with tracking spontaneous movement impulses but emphasizes non-directive witnessing over structured movement experiments.
AEDP four-state map	How do defenses, core affect, and core state unfold in therapy?	Practitioner	Clinical/Theoretical	FSD parallels AEDP’s core-state softening but relocates the map into the participant’s own somatic awareness.
Felt sense polyvagal model (FSPM)	From which autonomic state does a felt sense arise and where can it move?	Practitioner	Clinical/Theoretical	FSPM maps autonomic state; FSD maps the phenomenology of sensation within any given state, making the two complementary.
Integral somatic psychology (ISP)	How does guided expansion of emotion across body regions aid regulation?	Practitioner	Clinical/Practice-based	ISP directs expansion; FSD treats spreading and shifting as autonomous movements under non-attached attention.
CRM shift and stay	How can attention be shifted to more comfortable sensations for stabilization?	Practitioner	Practice-based/Community	CRM instructs attention shifts; FSD describes spontaneous shifting and softening when sensation is not redirected.
Somatic reappraisal (MABT)	How does sustained interoceptive attention support new meaning-making?	Practitioner and participant	Empirical/Practice-based	MABT documents outcomes of interoception; FSD provides a phenomenological topology of the somatic arc that may underlie such outcomes.
Felt sense dynamics (FSD)	How does sensation move when it is genuinely accompanied?	Participant (practitioner as teacher)	Phenomenological/Practice-based	Practice-based topology of the Five S’s arc and a transferable capacity for compassionate self-witnessing.

“Map held by” refers to the primary locus of framework knowledge during a session. “Evidence base” describes the primary epistemological foundation of each framework.

## The framework: felt sense dynamics and the five S’s

### Theoretical premise: sensation as dynamic process

Felt Sense Dynamics is grounded in the phenomenological insight, articulated most fully by Merleau-Ponty (2012), that we do not have bodies but are our bodies; the body is not the vehicle through which we experience the world but the very medium of experience itself. Emotion is not a mental event that causes bodily symptoms but a bodily event that may or may not reach linguistic articulation.

This premise is reinforced by contemporary neuroscience. Damasio’s (1994, 2003) somatic marker hypothesis holds that emotional responses originate as bodily signals that shape cognition before reaching conscious awareness. Barrett’s (2017) constructionist theory of emotion further complicates the picture: emotions are not biologically fixed categories waiting to be discovered but are actively constructed from bodily states, past experience, and contextual interpretation. Price and Hooven’s (2018) finding that sustained interoceptive attention causes sensation to “become more granular, vivid, and shift” provides direct empirical support for the core observation of the present framework: that sensation, when attended to, moves.

The core observation of Felt Sense Dynamics is that this movement follows a recognizable arc. The five phases of that arc are described below. They are not linear stages; a sensation may move through them in any order, skip phases, return to earlier ones, or remain at one phase for an extended period. They describe a topology of movement, not a prescribed sequence. “Compassionate curiosity without attachment” refers to an attentional stance that is warm, interested, and non-reactive, neither pushing sensation toward resolution nor retreating from its intensity. “Non-attached attention” means full, warm presence with sensation rather than distance from it. In practice, this stance is supported by trauma-informed structure and grounding, and is offered only within each participant’s window of tolerance, as discussed in the safety considerations below.

A common pattern in somatic practice is identifying where a feeling lives in the body without sustained attention to allow it to complete its arc. Identification and feeling are not the same thing. Many practitioners are trained to locate sensation, asking “where do you feel that?”, but the body’s process is short-circuited when attention moves on before the sensation has been given time to move. The difference is analogous to noticing the tickle of a sneeze without allowing it to complete; the sensation is contacted but not metabolized. Sustained, non-attached attention, held until sensation builds, peaks, and falls to a natural resting point, allows the energy of a feeling to be fully felt. This is what Felt Sense Dynamics attends to: not the location of sensation, but its sustained accompaniment through the full arc.

### Why accompaniment reveals what brief attention does not

Why would sustained, non-directive accompaniment reveal phenomenological dynamics that brief attentional contact does not? The question matters, because the answer is not simply that more time produces more data. Something qualitatively different occurs when attention rests with sensation long enough for the sensation to complete its own organizational logic.

Several converging frameworks illuminate this. At the neurophysiological level, interoceptive research suggests that sustained, non-evaluative attention allows bodily signals to become progressively more differentiated and permeable; less experienced as solid or fixed, more experienced as processes in motion (Garland, 2024; Price and Hooven, 2018). Research on pain tolerance specifically suggests that the capacity to remain with uncomfortable bodily sensation, rather than avoiding or managing it, functions as a protective factor rather than an amplifier of suffering. Decreased interoceptive awareness has been found to be a risk factor for the onset and chronicity of pain (Takaoka et al., 2023), while sustained mindful attention to bodily sensation has been associated with significant reductions in pain-related distress, increased emotional granularity, and improved capacity for self-regulation (Garland, 2024; Price and Hooven, 2021). What the body experiences as manageable expands when attention is brought to it with curiosity rather than aversion.

From the non-dual tradition, the mechanism is understood differently but compatibly. The evaluative overlay that makes sensation intolerable—the judgment that what is arising is bad, wrong, or dangerous—is what constrains its natural movement. When that overlay is suspended, as non-dual witnessing practice trains it to be, sensation is freed to be what it is rather than what we fear it might become. Gendlin’s Focusing arrives at a similar understanding from within phenomenological psychology: the invitation to stay with the felt sense, to resist the rush toward resolution, is precisely the gesture that allows the body to carry its implicit knowing forward into movement and meaning. Felt Sense Dynamics extends this shared insight by proposing a phenomenological topology of what unfolds when accompaniment is sustained.

### Boundaries of the framework

Before describing the Five S’s in detail, it is useful to state explicitly what Felt Sense Dynamics is not. It is not a diagnostic model; it does not categorize emotional states, symptom clusters, or psychological conditions. It is not a stage theory; the phases do not represent fixed developmental achievements, and the absence of any phase in a given session does not indicate pathology or failure. It is not an emotion taxonomy; it describes the movement of sensation, not the meaning or identity of feelings. It is not a treatment protocol; it does not prescribe specific interventions, session structures, or clinical procedures. It is not a replacement for existing somatic modalities, as the comparative analysis above demonstrates: it addresses a dimension of somatic experience that established frameworks have not explicitly mapped and is designed to complement rather than compete with them.

What Felt Sense Dynamics offers is a phenomenological topology: a descriptive map of the directions sensation tends to travel when genuinely accompanied. It is offered as a practice-based framework and as a starting point for empirical investigation, functioning as hypothesis-generating rather than as a formally tested clinical protocol. These distinctions matter for both appropriate use and the design of future research.

### Why five phases?

The topology of five phases was not predetermined. As described in the Practice-Based Origins section, the author attempted multiple organizational schemes before settling on this one. Fewer than five phases failed to capture the qualitative distinctions that participants themselves repeatedly described: the difference between the initial steadying of sensation and its subsequent intensification is phenomenologically distinct, as is the difference between spreading across body regions and shifting in quality or location. More than five phases introduced distinctions that were either not reliably observable or that belonged more accurately to the secondary dynamics described later in this paper.

The five phases also correspond loosely to five fundamental directions of somatic movement: sensation may hold its ground (Steadying), increase in intensity (Strengthening), expand across body regions (Spreading), change in quality or location (Shifting), or diminish in charge and urgency (Softening). These five directions do not exhaust all possible somatic events. The secondary dynamics described below arise frequently and carry their own significance. Even so, they describe the primary arc that sensation follows when accompanied without direction. They function as cardinal directions of felt experience: not the only movements, but the organizing ones.

### Scope, limitations, and safety considerations

This framework was developed primarily in grief retreat, accompaniment, and practitioner training contexts with adult participants in Western settings, including many licensed clinicians and somatic practitioners. Its applicability to other emotional domains, clinical populations presenting with severe dissociation, active psychosis, or complex trauma without professional support, non-Western somatic frameworks, and age groups beyond adults remains unknown and warrants careful adaptation and study.

The facilitation protocol assumes a baseline capacity for present-moment interoceptive awareness. It is not intended as a standalone intervention for individuals with significant destabilization, active self-harm risk, or unresolved complex trauma without appropriate clinical support. Practitioners are encouraged to assess each participant’s window of tolerance, monitor for signs of overwhelm or dissociation, and maintain clear referral pathways. These boundaries underscore that Felt Sense Dynamics is offered as a practice-based phenomenological framework that invites, rather than replaces, future empirical and clinical evaluation.

**Figure 1:**
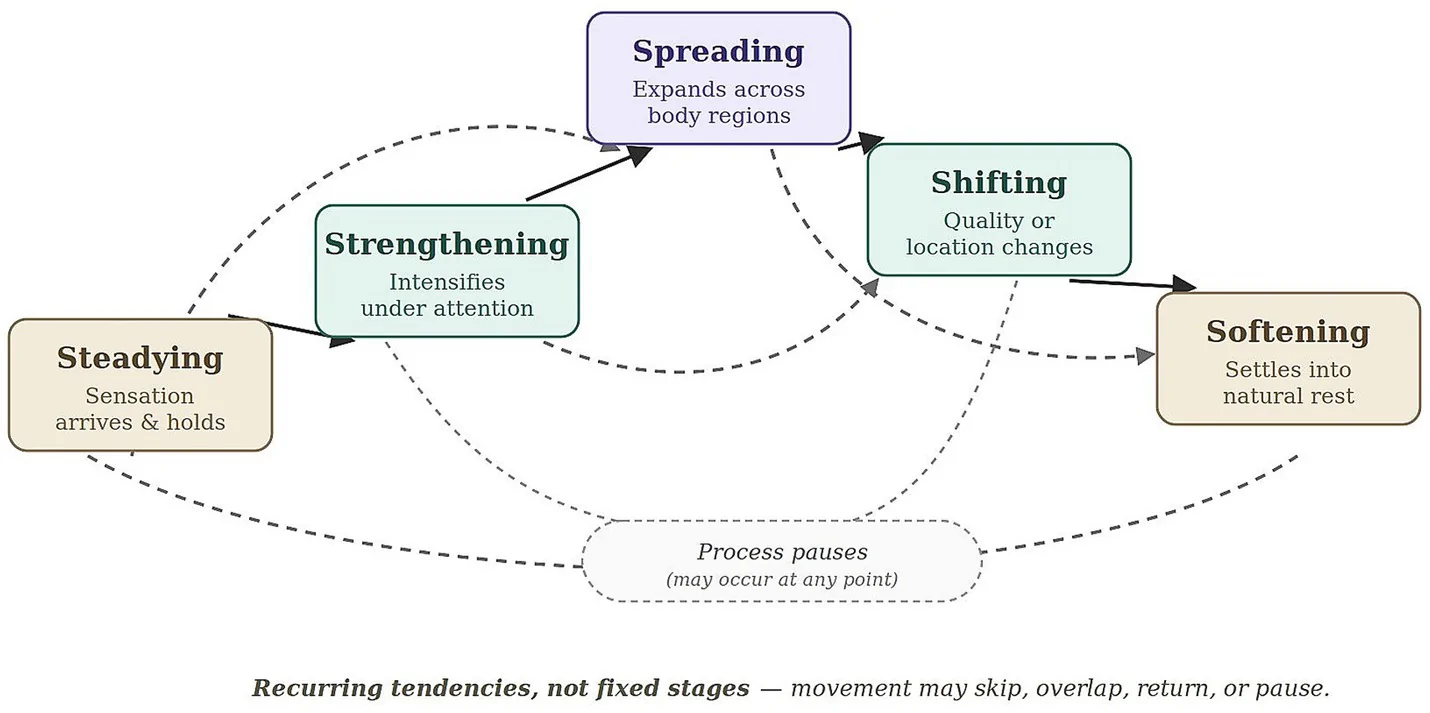
Schematic illustration of the felt sense dynamics arc, showing steadying, strengthening, spreading, shifting, and softening as recurring tendencies in the movement of sensation under sustained, non-attached attention.

The consistency of the five-phase topology across participants with widely varying emotional content, somatic backgrounds, and cultural contexts led the author to regard the topology as descriptively adequate rather than theoretically imposed. The topology is therefore intended as a parsimonious descriptive framework rather than a claim that all meaningful somatic events can be reduced to five categories. Future empirical work employing independent raters and structured coding protocols will be necessary to formally evaluate this claim. Figure 1 provides a schematic illustration of the Five S’s arc.

## The five S’s

### Steadying

When a sensation is first contacted in the body, it typically presents in a relatively stable form for the first several seconds of attention. This is the arrival state: the body has been noticed and holds its ground. In experiential terms, this is the moment of genuine contact; the practitioner and participant have arrived at what is present. The sensation says: I am here.

### Strengthening

As attention is maintained without interference, the sensation frequently intensifies. This is particularly pronounced with feelings that have been chronically denied, suppressed, or socially prohibited: grief, rage, shame, longing. The intensification is not a sign that something is going wrong; it is a sign that the sensation has finally been given genuine permission to exist. This phase often produces visible physiological changes: deepened breath, flushed skin, visible tension, or the early signs of tears. Price and Hooven’s (2018) observation that sustained interoceptive attention causes sensation to become “more granular and vivid” maps closely onto what is observed here. The practitioner’s task is to remain steady, communicating through presence that the participant’s experience is welcome.

### Spreading

Often following Strengthening, the sensation begins to spread through the body. What began as tightness in the chest may move into the throat, the jaw, the shoulders, the belly. The participant is invited to track this movement without trying to contain it. In the language of Body–Mind Centering (Cohen, 1993), this phase involves attending to how the sensation moves across different body systems—muscular, fascial, visceral—each of which may hold it differently.

### Shifting

Sensation frequently shifts in quality, texture, or location. A burning tightness may become a pulsing pressure; a heaviness may become a trembling. This phase often involves what Levine (1997) would recognize as the body beginning to complete an interrupted response: a movement impulse arising, a spontaneous sound, a breath of unexpected depth. Consistent with the Feldenkrais (1972) Method’s attention to movement exploration, when a sensation feels stuck, gentle exploratory movement can facilitate the Shift that the body is seeking.

### Softening

The arc of sensation, when held without interference, tends toward a natural resting point; not elimination but Softening. The sensation does not vanish so much as lose its urgency and demand for attention. Tears that have been allowed to come reach their natural end. Rage that has been tracked without action or suppression settles into something quieter. The participant frequently reports a sense of spaciousness, release, or unexpected calm. This is the moment Gendlin (1978) would recognize as the completion of the focusing process, and it arrives not because it was sought but because it was allowed.

## Illustrative practice observations

The following practice observations illustrate how the Five S’s can be discerned as a session unfolds. Identifying details have been altered to protect participant confidentiality.

### First observation

A participant, a woman in her late 60s whose husband of 30 years had died 2 years earlier, arrived for a session focusing on loneliness and emptiness. After grounded preparation, she was invited to bring a recent moment of loneliness to mind and locate where it arrived in her body. “Tightness in my chest,” she said. “Like something pressing from inside.” The sensation held its ground for the first several breaths, corresponding to Steadying.

As attention was sustained without intervention, she reported that it “feels like it’s squeezing harder now.” The tightness deepened, tears began to rise, and her breathing became slower and more effortful. The sensation then moved “up into my whole head and torso,” followed by “pressure behind my eyes.” Tears flowed freely. This progression illustrated Strengthening as sensation intensified under attention, Spreading as it moved across body regions, and Shifting as its quality transformed.

After remaining with this without any attempt to interpret or redirect it, she said, “It’s not gone, but it’s softer now.” Her whole body settled into a quality she described as “relaxed and a little lighter.” This corresponds to Softening, the diminishment of charge into a natural resting point.

In a second sequence within the same session, she tracked emptiness, which arrived as “tightness in the heart” and then shifted into anxiety—clamminess in the hands, racing thoughts—before moving again into tears and then gradually softening. Across both sequences, the experiential trajectory could be located within the Five S’s while also showing that individual arcs need not be linear or uniform. Nothing was directed. The practitioner tracked and accompanied. The participant’s body organized the process.

### Second observation

A second observation, drawn from a practitioner training context, illustrates how the arc operates across a different emotional domain and in a participant with prior somatic training. The participant, a licensed mental health professional, chose to work with anxiety arising from sustained responsibility for a friend in crisis. She located the feeling as a pressure pulling downward through her stomach. “Like something being dragged down,” she said. The sensation held its location for the first several breaths, corresponding to Steadying.

As attention was sustained without intervention, the pressure moved upward through her chest and into her throat, taking on a different quality: fuller and more resonant, less localized. Her breathing deepened. This illustrated Strengthening as the sensation intensified under attention and Spreading as it moved across body regions.

Without prompting, she reported: “It feels like it is loosening somehow. Not gone, but the pulling stopped.” The sensation appeared to shift from downward pressure to something more diffuse. She paused, then: “There is something sad underneath it.” This corresponds to Shifting, in which sensation changes quality or location, sometimes revealing layered emotional content beneath the presenting feeling.

The session concluded with the sensation softening into what she described as “a quiet kind of tiredness, but not a bad one,” corresponding to Softening.

This second observation illustrates several features of the framework. The participant, despite her clinical training and familiarity with somatic concepts, moved through the phases without directing or naming them in advance. Shifting revealed layered emotional content—grief beneath anxiety—without interpretive intervention. Together, these features underscore the framework’s core premise that sensation, when accompanied without direction, organizes its own movement. Comparable trajectories were observed repeatedly across practice settings, although the specific sequence, duration, and experiential qualities varied between individuals.

While this study does not propose a definitive neurophysiological mechanism for the Five S’s arc, several candidate mechanisms are consistent with the observed phenomenology. Autonomic regulation offers a first account: the Strengthening phase may reflect sympathetic activation as stored emotional material becomes accessible, while Softening may reflect a return toward ventral vagal states as the nervous system completes its processing arc, consistent with Porges’ (2011) polyvagal model. Interoceptive network modulation provides a complementary account: sustained, non-evaluative attention to bodily sensation may progressively activate and differentiate interoceptive representations, consistent with Price and Hooven’s (2018) finding that such attention causes sensation to become more granular and vivid before shifting, and with Garfinkel and Critchley’s (2013) evidence that interoceptive attention increases the salience and differentiation of bodily signals. At the level of meaning-making, Barrett’s (2017) constructionist framework suggests that sustained interoceptive attention may interrupt habitual emotion constructions and allow new conceptual resolutions to emerge from the body’s ongoing signaling. These accounts are not mutually exclusive and may each illuminate different dimensions of the same process. Formal testing of these mechanisms is a priority for future research.

### The sixth possibility: full stop and the tracking of dissociation

The framework recognizes a sixth possible event that may interrupt the arc at any point: a Full Stop, in which sensation abruptly ceases or the participant dissociates. Rather than treating this as a concern requiring immediate correction, Felt Sense Dynamics approaches it as a somatic event available for tracking. If the participant can observe what dissociation feels like in the body—the numbness, the fogginess, the sense of distance or unreality—that observation is valuable for integration. This reframing is supported by somatic trauma literature. Sensation, Image, Behavior, Affect, Meaning (SIBAM; Levine, 1997) is a channel model that describes the components of a complete somatic experience rather than the trajectory of sensation over time; it maps what experience is made of, where the Five S’s map how sensation moves. Within SIBAM’s framework, dissociation represents a fragmentation of experiential channels. Ogden et al.’s (2006) Sensorimotor Psychotherapy takes a compatible stance, positioning mindful awareness of dissociative states as a step toward their integration.

### The facilitation protocol

The Five S’s do not stand alone; they are embedded in a structured facilitation protocol developed through practice. The protocol consists of five elements: grounded preparation, emotion selection, memory activation, somatic tracking through the Five S’s, and pleasure anchoring. Regulated breathing serves as the transition point between each sequence. Many participants, in practice, come to rely less on the full five-part structure than on its central move: once a feeling is located in the body, staying with it to completion. This requires no elaborate preparation, only a willingness to find a time and space, in the moment or later, in which the sensation can be allowed to metabolize.

### Grounded preparation

Each session begins with a shared grounding practice: slow, diaphragmatic breathing, or the soft-belly breath. This invites the participant to arrive in the body before engaging any emotional material. This reflects both the Polyvagal-informed principle that the nervous system must be sufficiently regulated before activation is safe (Porges, 2011) and the Alexander Technique’s (Alexander, 1932) attention to postural and breath awareness as a foundation for authentic bodily experience. The participant is also invited to notice any area of the body that feels neutral or relatively at ease—an internal resource to return to throughout the session, consistent with CRM’s “Resourcing” skill (Miller-Karas, 2021) and SE’s emphasis on establishing safety before activation. For participants who find diaphragmatic breath focus activating, alternative grounding practices, including a slow body scan, sensory orientation to the immediate environment, or gentle bilateral weight-shifting, may be offered in place of or alongside the breath.

### Emotion selection and memory activation

Participants are invited to identify a challenging or significant feeling or emotional quality. Alternatively, they may begin with a troubling or unresolved situation, event, or memory, without even knowing what emotion underlies it; the somatic tracking process often clarifies the emotional content. They are then asked to bring the feeling or situation to mind, past or recent, allowing it to become present so that its affective and somatic dimensions are available for tracking. Participants do not need to narrate the memory in detail; a brief reference is sufficient. The instruction is to bring the memory consciously alive in the present moment, allowing it to become present rather than recalling it from a distance, so that its affective and somatic dimensions are available for tracking.

This approach is consistent with SE’s understanding that somatic processing requires sufficient activation to access stored material (Levine, 1997), while the instruction to reference rather than narrate keeps the participant present in their body rather than pulled into cognitive re-experiencing. It also creates the conditions Price and Hooven (2018) identify as necessary for somatic reappraisal: a specific internal focus with enough emotional charge to generate meaningful interoceptive data.

### Somatic tracking through the five S’s

Once the emotion is present and located in the body, participants are guided to attend to its somatic expression with compassionate curiosity, without attachment. The practitioner invites them to describe the sensation—its location, texture, temperature, weight, movement—without interpreting or seeking to change it. The practitioner tracks the participant’s descriptions for signs of movement through the Five S’s, mirroring and accompanying rather than directing.

The question of whether to name an emotion before tracking its somatic expression is addressed flexibly. Consistent with Barrett’s (2017) finding that emotional categories are constructed rather than discovered, the framework holds naming lightly: a name may be offered as a starting orientation, but the practitioner recognizes that it is provisional. A participant who begins by naming “anger” may find, through tracking, that what is present is closer to grief, shame, or longing, or some compound of all three that resists a single label. This approach is consistent with Gendlin’s (1978) resonance-checking, testing whether a word fits the felt sense, and extends to include the possibility that no word may fit, and that the sensation can be tracked without one.

### Macro-pendulation and pleasure anchoring

A single session may move through two or three challenging emotional sequences, each separated by a return to soft-belly breath. This structure constitutes what the author terms macro-pendulation. Levine’s classical SE pendulation oscillates moment-to-moment within a single sensory experience, moving in close oscillation between a distressing sensation and a neutral or resourced bodily state. Macro-pendulation operates at a different scale: it oscillates between complete emotional cycles, each of which is tracked through to Softening before the return to regulated breath.

The distinction is one of rhythm rather than principle. Both approaches pursue the same regulatory goal, preventing the nervous system from becoming overwhelmed while allowing sufficient activation for genuine processing, but through different temporal structures. SE’s moment-to-moment pendulation titrates activation within a single experience; macro-pendulation completes one experience before activating the next. The effect is cumulative and, in the author’s practice-based observation, reliably effective across many sessions. Participants report feeling significantly better at the close of a session not because difficult material was avoided but because it was moved through rather than around. The breath return between sequences ensures that activation does not accumulate beyond the participant’s window of tolerance (Siegel, 1999), while the completion of each arc before returning to breath reinforces the nervous system’s capacity to tolerate and metabolize emotional intensity.

The session concludes with somatic tracking of a pleasurable or positive feeling, following the same protocol. This pleasure anchoring ensures the session closes with the nervous system in a regulated, ventral vagal state (Porges, 2011) and expands the participant’s somatic vocabulary beyond difficult feelings.

### The core five and the extended dynamics

The five phases described above, Steadying, Strengthening, Spreading, Shifting, and Softening, represent the core topology of sensation movement as observed across the author’s practice. A claim of the framework worth stating explicitly is that no complete arc has yet been observed that could not be described using one or more of these five phases. At present, this is a practice-based observation that calls for systematic empirical testing.

The Five S’s are not a sample of possible somatic movements but the primary observed directions sensation travels: a working topology of the arc, not an exhaustive taxonomy. The framework also recognizes a set of secondary somatic conditions that arise as modifiers or interruptions to the primary arc, which might be termed the weather patterns of sensation. Where the Five S’s describe the cardinal directions of somatic movement, these secondary dynamics describe atmospheric conditions that arise around and between those movements. They include oscillation between states (Sway), suspension that conceals resistance or freeze beneath apparent stillness (Stall), fragmented or erratic sensation without clear location (Scatter), collapse into over-intensification (Sink), sudden puncture or flash of sensation (Spike), and the subtle emergence of sensation beginning to surface before entering the primary arc (Surface). The full Stop described in the preceding section also belongs to this secondary register.

These weather patterns are real, substantively significant, and frequently observed in complex or layered grief work. A full account of their phenomenology, their relationship to the primary arc, and their facilitation implications is beyond the scope of this paper. They constitute a meaningful direction for future elaboration of the framework. What the present paper establishes is the primary architecture: the Five S’s as the core within which all secondary dynamics arise and to which they eventually return.

### Trauma-informed structure and the question of retraumatization

A rigorous trauma-informed evaluation of any somatic protocol must address the question of retraumatization risk: whether sustained engagement with painful sensation, without moment-to-moment pendulation, risks overwhelming a participant’s regulatory capacity. This concern is legitimate and warrants a direct response.

The facilitation protocol of Felt Sense Dynamics incorporates four structural elements that together constitute a trauma-informed architecture. First, grounded preparation—the soft-belly breath and neutral body resource established before any emotional material is engaged—ensures that participant’s nervous system begins from a regulated, ventral vagal state (Porges, 2011). Second, participants are guided to bring a memory to mind and track its somatic expression in the present body, rather than re-enter or relive the memory itself. This distinction, between present-moment somatic tracking and experiential reliving, is critical: the participant remains anchored in the present body even while emotionally activated, which substantially reduces the risk of flashback and overwhelm. Present-moment anchoring is offered here as one trauma-informed titration strategy consistent with the framework’s structure, not as a universal prescription or as empirically superior to all forms of reliving work. Third, the framework’s non-directive stance means the pace is set by the participant’s body rather than the practitioner’s agenda. Sensation that intensifies beyond a participant’s capacity to remain present may elicit a full Stop, a dissociative interruption that the framework treats as trackable rather than as a failure. In many cases, such interruptions can be understood as attempts at internal regulation or protection, even when they limit connection or functioning. Fourth, every session closes with pleasure anchoring, ensuring the participant’s nervous system returns to a ventral vagal state before the session ends (Porges, 2011; Levine, 1997).

Across many years of individual sessions in grief retreat, accompaniment, and educator training contexts, the author has not knowingly observed retraumatization as defined by the field, emotional flooding, collapse, dissociation to the point of loss of present-moment contact, or prolonged post-session destabilization, though formal outcome tracking has not been undertaken and this observation is limited to the contexts described. This practice-based observation is consistent with the framework’s structural design. The protocol does not require pendulation at the moment-to-moment level because its other structural elements—grounded preparation, present-moment anchoring, internal pacing, and pleasure anchoring—accomplish the same regulatory function through different means. Practitioners with clinical training working with participants with significant trauma histories may, at their discretion, incorporate moment-to-moment pendulation within individual sequences as an additional titration strategy, consistent with SE protocol.

## Theoretical considerations

### Non-duality and the witness perspective

A distinctive feature of Felt Sense Dynamics is its grounding in non-dual philosophy as a somatic and contemplative orientation. In many Western somatic and psychological frameworks, emotional pain is approached through a dualistic lens: the feeling is something to be understood, processed, resolved, in some sense overcome. The framework proposed here makes a different move. Drawing on contemplative traditions in which witnessing awareness is understood as prior to and more fundamental than the content of experience, it invites both practitioner and participant into a mode of presence that is neither attached to the sensation nor retreating from it. Here, non-duality is used pragmatically to describe a shift in attentional stance rather than as a metaphysical claim.

The non-dual witness does not rise above grief; it enters grief fully enough that grief can move.

A further distinguishing orientation of Felt Sense Dynamics is that this witnessing capacity is understood as learnable and transferable. Many somatic frameworks position the practitioner as the holder of specialized knowledge—the one who identifies the state, tracks the nervous system, applies the intervention. Felt Sense Dynamics positions the practitioner differently: as a teacher of a capacity rather than a holder of a dependency. The Five S’s are taught so that participants can eventually use them independently: in a session, in a difficult conversation, in a moment of rising anger or unexpected grief. This orientation aligns with a broader movement in contemporary mental health toward scalable, self-directed tools that democratize access to embodied self-knowledge. The goal is not only effective sessions but a more deeply inhabited life between and beyond them.

### Grief as primary context

Although Felt Sense Dynamics is applicable across the full range of emotional experience, it was developed primarily in the context of grief work. Grief presents a particular challenge to somatic approaches as it is often chronic rather than acute, layered across multiple losses and time periods, and socially constrained in ways that make embodied expression difficult. Participants in grief frequently present with somatic symptoms that medicine cannot locate, pain without clear origin, or physical exhaustion unresponsive to rest.

The framework’s non-teleological orientation, its refusal to organize around outcome or resolution, is particularly well-suited to grief. Grief does not resolve; it transforms. The mourner does not move through grief toward its end but learns, gradually, to live within it differently. Felt Sense Dynamics offers a practice for that ongoing inhabitation: not a technique for processing grief toward completion, but a way of remaining with it long enough for it to move.

### Epistemological basis: organic inquiry and practice-based knowledge

Felt Sense Dynamics was developed inductively through sustained, attentive somatic practice across many years, in the tradition of what Clements et al. (1998) describe as organic inquiry—a research methodology in which the investigator’s embodied and experiential engagement with a phenomenon is understood as a legitimate and primary mode of knowing.

Practice-based evidence, defined as knowledge derived from the accumulated observations of skilled practitioners across many cases, has increasingly been recognized as a legitimate complement to evidence-based practice in the psychological and somatic fields (Barkham and Margison, 2007; Stiles et al., 2006). The Five S’s emerged not from a theory that was then tested in practice, but from a pattern that presented itself repeatedly in practice and then sought theoretical articulation. This distinction is methodologically relevant for interpreting the framework’s claims.

Rather than presenting controlled empirical validation, this paper formalizes consistent observational findings that can be operationalized and tested in future research. The Five S’s and the facilitation protocol can be translated into replicable procedures, and outcomes can be measured. The author invites collaboration with researchers in somatic psychology, body psychotherapy, and grief studies to undertake that work. Because these observations emerged through practice rather than prospective study, they should be understood as hypothesis-generating rather than hypothesis-confirming.

As with any practice-based phenomenological framework, the observations underlying Felt Sense Dynamics are subject to influences that cannot yet be disentangled within the present practice-based design. Practitioner expectation is among the most significant: a practitioner who anticipates a five-phase arc may attend more readily to confirming evidence and less readily to deviations. Demand characteristics in a guided setting may also shape participant reports, as participants may unconsciously orient their descriptions toward what they perceive the practitioner expects. Natural habituation and adaptation processes within the nervous system may produce sensation changes that are independent of any framework-specific mechanism. Additionally, the quality of the relational field, the degree of trust and felt safety between practitioner and participant, may itself be a variable shaping whether and how sensation moves. Future empirical studies employing independent raters, structured protocols, and control conditions will be necessary to disentangle these contributions from the framework-specific effects the present paper proposes.

The framework was developed primarily in Western contexts, with additional informal exposure through cross-cultural practice, and should not be assumed to generalize universally without further examination. Cultural frameworks vary significantly in how they conceptualize the relationship between body, emotion, and selfhood, and these differences are likely to shape both how sensation is experienced and how it is reported. Cross-cultural adaptation and empirical study are warranted to clarify how the framework functions across cultural settings and to support broader claims.

### Preliminary observational structure and future empirical directions

The practice-based observations described above provide a preliminary structure that can be translated into operational criteria for empirical study of the Five S’s. The consistency of the observed phase transitions across diverse emotional content, participant backgrounds, and practice contexts suggests that the Five S’s are suitable for systematic investigation. The following sections outline one way these criteria could be specified and tested in formal research.

### Operationalization

For the Five S’s to be empirically studied, reliable phase identification is necessary. Each phase can be operationally defined by participant-reported descriptors. Steadying is indicated by reports of stable, localized sensation with no reported change: “it’s still there,” “it’s holding.” Strengthening is indicated by reports of increasing intensity, pressure, or physiological activation: “it’s getting stronger,” “it’s more intense,” “it’s building.” Spreading is indicated by reports of expansion across body regions: “it’s moving upward,” “it’s spreading into my chest,” “it’s everywhere now.” Shifting is indicated by reports of qualitative change in texture, location, or kind: “it changed,” “it’s pulsing now,” “it jumped to my throat,” “something shifted.” Softening is indicated by reports of diminishing intensity, release, or ease: “it’s fading,” “it’s softer,” “It is dissipating.” These descriptors are drawn directly from participant reports across many sessions and represent the language that has most reliably marked phase transitions in practice-based observation.

### Falsifiability

A phenomenological topology of this kind makes implicit predictions that are, in principle, testable and potentially disconfirmable. The framework would require substantial revision if independent coders cannot reliably identify phases above chance, if observed sequences are predominantly random rather than arc-structured, if full arcs through Softening do not relate to greater reductions in perceived emotional intensity, or if participants themselves cannot distinguish the phases as phenomenologically distinct. The framework does not claim that every session will produce a complete arc—the secondary dynamics and Full Stop are explicitly recognized—but it does claim that the five phases describe the primary directions sensation travels when accompanied without direction, and that this claim is empirically tractable.

### Specific hypotheses

Three hypotheses are proposed for initial empirical investigation. First, that the five phases are reliably identifiable in session transcripts by independent raters trained in the operational descriptors above, with inter-rater reliability exceeding chance. Second, that completion of the full arc through Softening predicts a greater participant-reported reduction in emotional intensity, as measured by pre-post self-report, than sessions in which the arc was interrupted or incomplete. Third, that structured facilitation using the Five S’s produces greater improvements in interoceptive awareness, as measured by the Multidimensional Assessment of Interoceptive Awareness (MAIA; Mehling et al., 2012), than unstructured somatic tracking over a comparable period.

### Future research agenda

Beyond these initial hypotheses, the framework opens a broader empirical agenda. Coding studies could establish inter-rater reliability for phase identification and examine the frequency and sequencing of phases across different emotional domains, populations, and facilitation contexts. Physiological measurements, including heart rate variability, skin conductance, and respiratory patterns, could be time-aligned with participant-reported phase transitions to examine autonomic correlates of the arc. Studies examining different emotional domains beyond grief would test whether the topology is as general as practice-based observation suggests. Cross-cultural studies are needed to examine how cultural frameworks shaping the conceptualization of body, emotion, and sensation affect both phenomenological reports and the structure of the arc. Clinical population studies, with participants presenting with complex trauma histories, alexithymia, or conditions affecting interoceptive awareness, would examine what modifications the framework may require. Finally, training studies examining whether practitioners who learn to teach the Five S’s produce better participant outcomes than those using unstructured somatic tracking would provide evidence relevant to the framework’s clinical utility. The research agenda proposed here represents the pathway from that observation toward the formal understanding the field deserves. The Five S’s were not invented and then observed; they were observed repeatedly before they were named.

## Conclusion

When held with patient, non-attached attention, sensations move. This is the central observation of Felt Sense Dynamics. It is simple in one sense: practitioners across somatic traditions have long known that sensation, when not suppressed or forced, tends to find its own natural resting point. It is consequential in another, because naming the arc of that movement, identifying the Five S’s as a recognizable topology, offers practitioners a map of previously unnamed terrain.

The map does not prescribe the journey. Participants move through the Five S’s in their own order and at their own pace, without a predetermined destination. What the framework provides is not a protocol for directing sensation but an orientation for accompanying it, a way of remaining present with what is happening in the body without needing it to be otherwise. This distinguishes it from directive models like CRM’s Shift and Stay and ISP’s emotional embodiment protocol, and from relational models like AEDP whose vehicle of change is the therapeutic relationship rather than witnessing attention. It aligns with the witnessing orientation of focusing and the tracking orientation of Somatic Experiencing, while extending both by naming what tracking and witnessing, when sustained, tend to reveal. It complements rather than competes with Winhall’s FSPM, which maps the autonomic context of felt sensing, and with Price and Hooven’s somatic reappraisal framework, which maps its cognitive outcomes. Each addresses a different dimension of the same somatic process; Felt Sense Dynamics attends to the arc of sensation itself.

For grief practice, this orientation is particularly apt. Grief rarely resolves; it reorganizes. In many grief contexts, the question is not how to bring feeling to an end but how to live within it without collapse, chronic constriction, or dissociation. By offering a repeatable way to track grief’s somatic arc from Steadying through Softening, Felt Sense Dynamics supports mourners in remaining with difficult affect long enough for it to move, while honoring that movement as ongoing rather than final.

In grief retreat contexts, this has included participants facing what they described as “unimaginable” losses, including multiple child deaths and discovering loved ones deceased. Many arrived convinced that certain scenes or memories could not be revisited without collapse. Working somatically with these experiences—locating where they arrive in the body, accompanying the sensations through their arc, and closing with regulated states—has repeatedly enabled participants to feel what they had feared was unfeelable and to remain present with their “worst” pain. Several have described a surprising sense of empowerment in realizing that they can accompany these experiences rather than avoid them, and that their bodies can move through the arc without breaking. This practice-based observation suggests that one of the framework’s gifts, particularly in grief, is an experiential sense of agency in relation to suffering that had previously felt unapproachable.

For somatic education and practitioner training, the framework provides a teachable structure that participants can internalize and carry independently, shifting some of the core competencies of somatic work—tracking, witnessing, and staying—from practitioner-held expertise to participant-held capacity. This aligns with a broader movement in mental health toward scalable, self-directed tools that democratize access to embodied self-knowledge.

The practice-based nature of the framework invites and requires further empirical work rather than replacing it. The Five S’s can be operationalized for independent coding of session transcripts; the presence and completion of arcs can be related to changes in emotional intensity, interoceptive awareness, and functional outcomes; the facilitation protocol can be compared with unstructured tracking or other somatic interventions. Future studies may further examine how the arc operates across diverse emotional domains, cultural contexts, and clinical populations with more complex trauma histories, and identify what modifications, if any, are needed in those settings.

## Data Availability

The original contributions presented in the study are included in the article/supplementary material, further inquiries can be directed to the corresponding author.
